# Efficient photocatalytic degradation of gaseous acetaldehyde over ground Rh–Sb co-doped SrTiO_3_ under visible light irradiation

**DOI:** 10.1039/c7ra11337d

**Published:** 2018-01-31

**Authors:** Yuichi Yamaguchi, Sho Usuki, Kenji Yamatoya, Norihiro Suzuki, Ken-ichi Katsumata, Chiaki Terashima, Akira Fujishima, Akihiko Kudo, Kazuya Nakata

**Affiliations:** Research Institute for Science and Technology, Photocatalysis International Research Center, Tokyo University of Science 2641 Yamazaki Noda Chiba 278-0022 Japan nakata@rs.tus.ac.jp; Department of Applied Biological Science, Faculty of Science and Technology, Tokyo University of Science 2641 Yamazaki Noda Chiba 278-0022 Japan; Department of Applied Chemistry, Faculty of Science, Tokyo University of Science 1-3 Kagurazaka Shinjuku-ku Tokyo 162-8601 Japan

## Abstract

A visible-light-responsive Rh–Sb co-doped SrTiO_3_ photocatalyst (STO:Rh,Sb) *via* a solid-state reaction was successfully developed, following pulverization by using ball-milling. The prepared STO:Rh,Sb exhibited a large surface area and showed efficient photocatalytic degradation of acetaldehyde. The photocatalytic activity of STO:Rh,Sb ground for 60 min exceeded that of STO:Rh ground for 60 min (photocatalyst doped without antimony), indicating that doped antimony plays an important role in suppressing the Rh^4+^, which works as a recombination center, in STO:Rh,Sb. Furthermore, the photocatalytic performance of STO:Rh,Sb ground for 60 min was sustained over 3 cycles, confirming the chemical stability of the photocatalyst. Therefore, ground STO:Rh,Sb has the potential to be applied to environmental remediation under visible light irradiation.

## Abstract 2

A visible-light-responsive Rh–Sb co-doped SrTiO_3_ photocatalyst (STO:Rh,Sb) *via* a solid-state reaction was successfully developed, following pulverization by using ball-milling.

## Graphical abstract

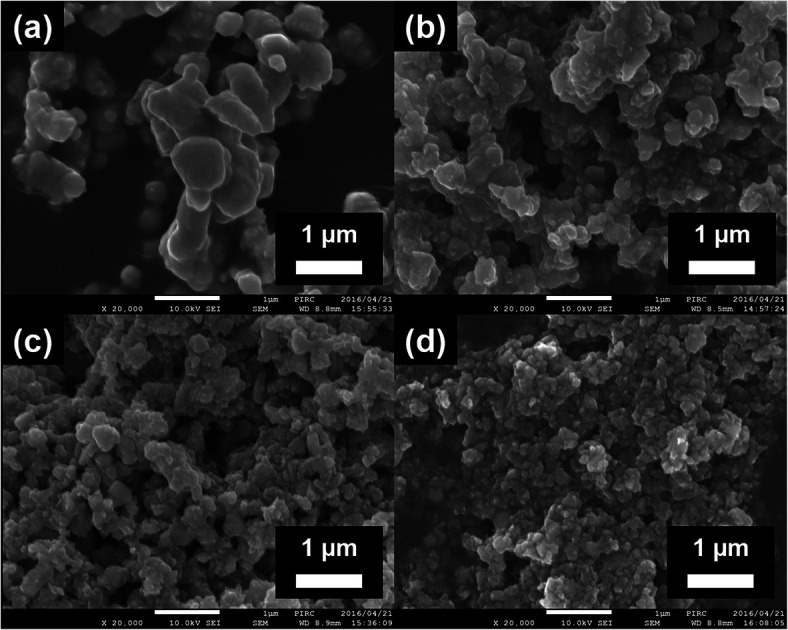

## 1. Introduction

Photocatalysts have been widely applied for hydrogen production from water and for environmental purification by light irradiation.^1–15^ TiO_2_ is a representative UV light-responsive photocatalyst which exhibits good photocatalytic performance.^16^ However, it is activated by UV light, which comprises only 3–5% of the solar spectrum. Therefore, the usability of TiO_2_ in solar applications is limited, and a photocatalyst responsive to visible light is strongly required. Doping with transition metals has been often attempted in order to extend the range of light absorption to visible light, and a metal doped TiO_2_ photocatalyst responsive to visible light was developed by various research groups.^17–21^ In addition, so far, numerous metal-doped SrTiO_3_ (Cr-, Ni-, Ir-, Mn-, Ru-, Ta-, and Rh-doped) photocatalysts that respond to visible light have been developed.^21–30^ Rh-doped SrTiO_3_ (STO:Rh) has gained special attention as a novel p-type metal oxide semiconductor photocathode and photocatalyst, and it efficiently evolves hydrogen from water under visible light irradiation.^24,31–33^ Overall water splitting has been achieved by combining Z-scheme photocatalytic systems with BiVO_4_ (an oxygen evolution photocatalyst)^34–42^ and STO:Rh (a hydrogen evolution photocatalyst).^43–46^ In contrast, the photocatalytic performance of STO:Rh for degradation of environmental pollutants is almost negligible because of its small surface area. In the recent years, STO:Rh with a large surface area was successfully prepared by ball-milling and its applicability to environmental remediation was demonstrated.^47,48^ Specifically, ground STO:Rh showed the selective anti-phage performance even in the presence of bacteria under visible light irradiation although TiO_2_ photocatalysis did not show,^48^ which indicates that ground STO:Rh has a unique visible-light-responsive photocatalyst for environmental application. Thus, it is of significance in order to investigate the photocatalytic activity of metal doped STO photocatalyst for environmental remediation.

Doping improves the response of STO to visible light, but often forms recombination centers between the photogenerated electrons and holes, resulting in low photocatalytic performance.^2^ Charge compensation by co-doping improves the photocatalytic performance by suppressing the recombination center.^20–23,27,49–51^ Interestingly, STO:Rh,Sb can evolve oxygen from water containing a sacrificial agent in photocatalytic water splitting, whereas STO:Rh cannot.^49–52^ In STO:Rh, the rhodium is doped as Rh^4+^ at the Ti^4+^ sites, forming recombination centers. In contrast, antimony is doped as Sb^5+^ in STO:Rh,Sb, forming Rh^3+^ by charge compensation. Antimony doping plays an important role in suppression of recombination centers.^2^

Herein, the photocatalytic degradation of acetaldehyde was evaluated using a STO:Rh,Sb photocatalyst responsive to visible light prepared by a solid-state reaction and ball-milling. The optimal grinding time and doping amounts of Rh and Sb were also investigated. To assess the effect of antinomy doping, the photocatalytic performances of optimized STO:Rh,Sb and ground STO:Rh were compared. The chemical stability of STO:Rh,Sb was examined by evaluating its photocatalytic degradation of acetaldehyde over three cycles.

## 2. Experimental

### 2.1 Preparation of photocatalyst

STO:Rh and STO:Rh,Sb were synthesized *via* a conventional solid-state reaction.^24^ The starting materials were SrCO_3_ (99.9%, Kanto Chemical Co., Inc., Japan), TiO_2_ (99.9%, Soekawa Chemical, Japan), Rh_2_O_3_ (95%, Wako Pure Chemical, Japan), and Sb_2_O_3_ (98%, Nacali Tesque Inc., Japan). These chemicals were mixed with a small amount of methanol (99.8%, 5 mL) at the required ratios in an aluminum crucible to obtain Sr/Ti/Rh/Sb = 1.07 : (1.00 − 2*x*) : *x* : *x*. The mixed powder was calcined in air at 900 °C for 1 h and then at 1100 °C for 10 h. The resulting powder (1.0 g) was transferred to a 45 mL container containing ultrapure water (5.0 mL) and zirconia balls (10.0 g; diameter = 1.0 mm) for milling. The powder was milled at 800 rpm for different specified times (30, 60, and 90 min) in a ball-milling device (Fritsch Pulverisette 7). Finally, the ground particles were collected by filtration, washed with ultrapure water, and dried at 60 °C for 24 h. In the present report, the samples are denoted STO:Rh(*y*%),Sb(*y*%), where *y*% refers to the quantity of the doped Rh and Sb.

### 2.2 Characterization of photocatalyst

The morphologies of the prepared photocatalysts were observed using a field emission scanning electron microscope (FE-SEM; JEOL-7600F, 10 kV). The crystal structures of the prepared photocatalysts were determined by X-ray diffraction (XRD; RIGAKU Ultima IV) using Cu Kα radiation. A UV-visible spectrometer (JASCO V-670) was used to obtain the diffuse reflectance spectra; the reflection data were converted into absorbance values using the Kubelka–Munk formula. The specific surface areas of the photocatalysts were determined by the Brunauer–Emmett–Teller method and were analyzed using a gas adsorption and desorption (MicrotracBEL BELSORP-max). Qualitative analysis for the elemental components of the photocatalyst was carried out using transmission electron microscope (TEM; JEM-2100F, 200 kV) equipped energy-dispersive X-ray (EDX).

### 2.3 Evaluation of photocatalytic oxidative degradation of acetaldehyde

The photocatalyst powder (0.3 g) was placed in a glass Petri dish (diameter: 35 mm), which was transferred to a 500 mL closed glass reactor. Air at 50% humidity was flowed into the reactor. Prior to the evaluation of the photocatalytic performance, the photocatalyst was illuminated under a 200 W Xe lamp (100 mW cm^−2^) without a cutoff filter to decompose any organic contaminants adsorbed on its surface. The photocatalyst was then irradiated with visible light at 100 mW cm^−2^ under a Xe lamp with an L-42 cutoff filter (*λ* > 420 nm) and atmospheric condition at 25 °C. The initial acetaldehyde concentration was adjusted to *ca.* 150 ppm. The concentrations of acetaldehyde and carbon dioxide were measured using a gas chromatograph (GC; Shimadzu GC2014, column; Porapak Q) with a flame ionization detector equipped with a methanizer, which converts the carbon dioxide to flammable methane. To calculate the concentration of acetic acid formed by an intermediate of the photocatalytic oxidative degradation process, the photocatalyst was washed with water (10 mL) *via* sonication for 5 min after the photocatalytic reaction. After filtration, the concentration of acetic acid in the wash water was determined *via* high-performance liquid chromatography (HPLC; Hitachi Chromaster) using a UV detector (Hitachi Chromaster 5410) at a wavelength of 210 nm. The HPLC measurements were obtained under the following conditions; column 250 × 4.6 mm (Hitachi LaChrom 2 C18), column temperature 25 °C, mobile phase 85% ethanol/15% water (v/v), and flow rate 1.0 mL min^−1^. The reaction rate coefficient (min^−1^) was determined as the slope of log(*C*_0_/*C*) *vs.* time (where *C*_0_ and *C* are the acetaldehyde concentrations at the initial irradiation time (0 h) and at time *t* during the irradiation, respectively).

## 3. Results and discussion

### 3.1 Characterization of pristine and ground STO:Rh,Sb

**Fig. 1:**
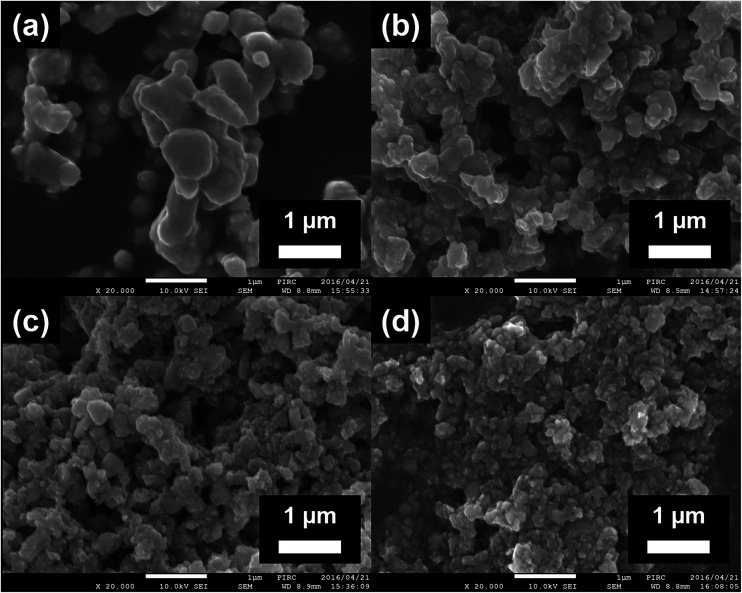
FE-SEM images of (a) the surfaces of pristine STO:Rh(1.0%),Sb(1.0%) and STO:Rh(1%),Sb(1%) ground for (b) 30 (c), 60 (d), and 90 min at 800 rpm.

Fig. 1 depicts the FE-SEM images of pristine STO:Rh(1%),Sb(1%) and STO:Rh(1%),Sb(1%) ground for 30, 60, and 90 min at 800 rpm. The sizes of pristine STO:Rh(1%),Sb(1%) particles were large (*ca.* 1–3 μm) with small surface area (2.0 m^2^ g^−1^), ascribed to sintering at high temperature (Fig. 1a). Ball-milling reduced the particle size of STO:Rh(1%),Sb(1%) and increased its surface area to 30, 42, and 47 m^2^ g^−1^ after grinding for 30, 60, and 90 minutes, respectively (Fig. 1b–d).

**Fig. 2:**
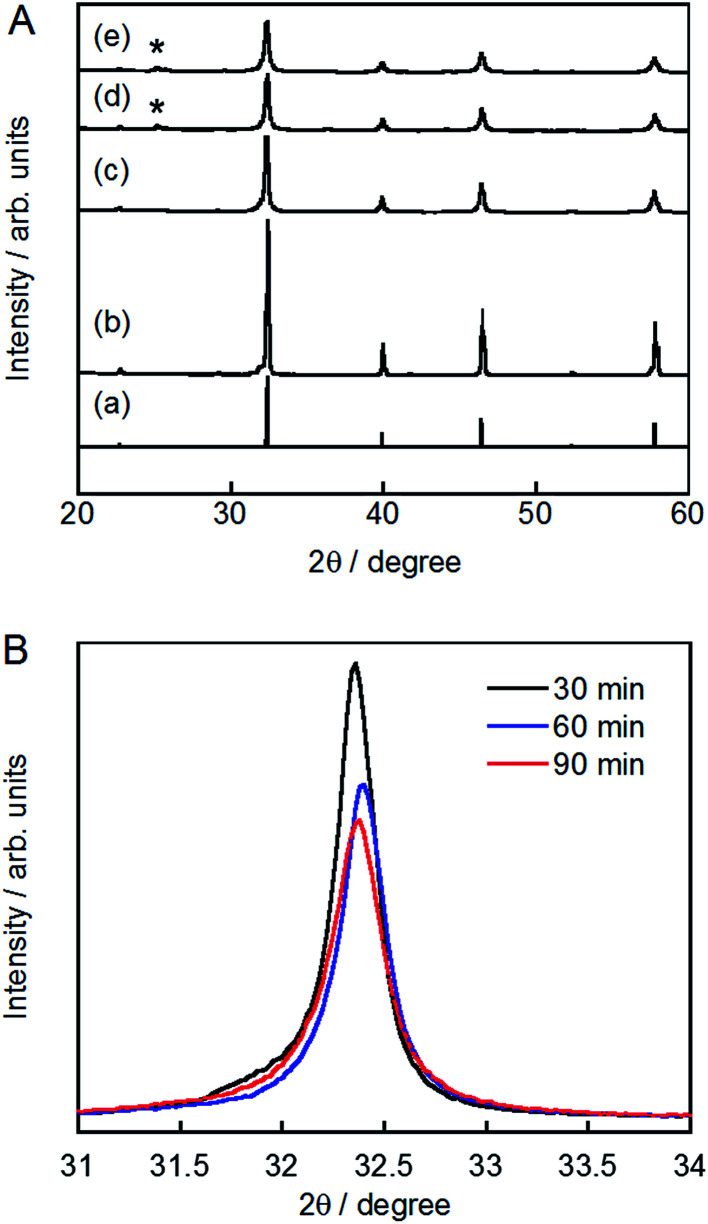
(A) X-ray diffraction patterns of (a) cubic SrTiO_3_ (JSPDS No. 01-073-0661), (b) pristine STO:Rh(1%),Sb(1%) and STO:Rh(1%),Sb(1%) ground for (c) 30, (d) 60, and (e) 90 min (*: anatase TiO_2_). (B) Enlarged peaks of STO:Rh(1%),Sb(1%) ground for 30, 60, and 90 min at 800 rpm.

**Fig. 3:**
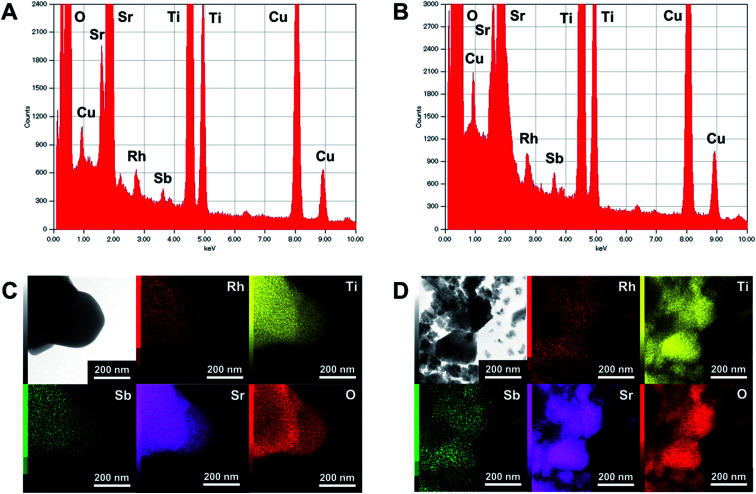
TEM/EDX spectra of (A) pristine STO:Rh(1%),Sb(1%) and (B) STO:Rh(1%),Sb(1%) ground for 60 min, and EDX mapping of (C) pristine STO:Rh(1%),Sb(1%) and (D) STO:Rh(1%),Sb(1%) ground for 60 min.

The XRD patterns of pristine and ground STO:Rh(1%),Sb(1%) were assigned to cubic STO (Fig. 2A). The peaks around 25° and 36.5° appearing at longer grinding times are inconsistent with the STO peaks and were assigned to anatase TiO_2_ and SrCO_3_, respectively. Ball-milling induces a crystalline phase transformation of STO to anatase TiO_2_ and SrCO_3_, which was attributed to the reaction between SrTiO_3_ and atmospheric CO_2_.^53^ Additionally, the peaks of ground STO:Rh,Sb were broadened by the grinding process, with the increase of the value of the full width at half maximum, as shown in Fig. 2B. The longer the grinding time should cause the decrease in particle size as seen in the FE-SEM images (Fig. 1). Fig. 3 depicts the TEM/EDX spectra and EDX mapping of pristine STO:Rh(1%),Sb(1%) and STO:Rh(1%),Sb(1%) ground for 60 min, which revealed that a small amounts of dopants of Rh and Sb were successfully detected and uniformly present in both STO:Rh and ground STO:Rh,Sb particles.

**Fig. 4:**
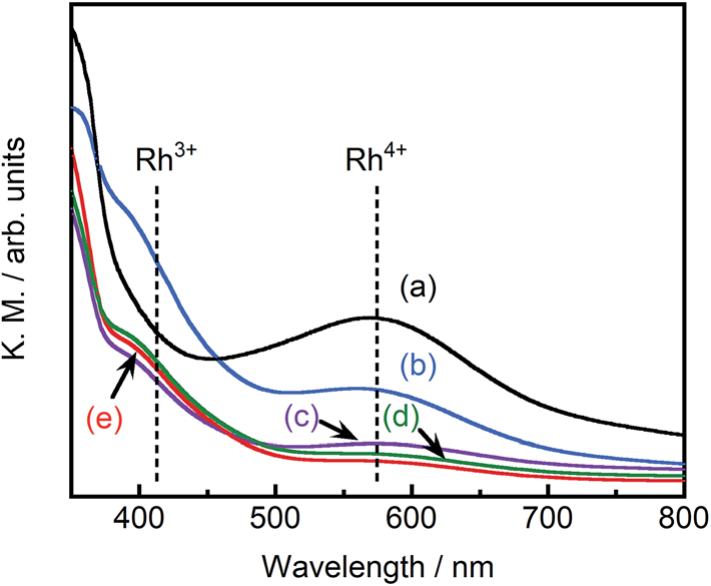
Diffuse reflectance spectra of (a) pristine STO:Rh(1%), (b) pristine STO:Rh(1%),Sb(1%) and STO:Rh(1%),Sb(1%) ground for (c) 30, (d) 60, and (e) 90 min.

Fig. 4 depicts the diffuse reflectance spectra of pristine STO:Rh(1%) and pristine ground STO:Rh(1%),Sb(1%). The measured values of absorbance at 580 and 420 nm are due to electronic transitions from the valence band of STO to an unoccupied d state of Rh^4+^ and from an occupied state of Rh^3+^ to the conduction band of STO, respectively.^24,32^ In ground STO:Rh, the 580 nm absorption related to Rh^4+^ was stronger than the 420 nm absorption related to Rh^3+^. In STO:Rh,Sb, the absorptions related to Rh^4+^ and Rh^3+^ were lower and higher, respectively, from those of STO:Rh. Rh^4+^ doping at Ti^4+^ sites in STO:Rh provides charge compensation,^24,31^ whereas doping with Sb stabilizes the Rh valence state as Rh^3+^. In this doping scheme, Rh^4+^ formation is suppressed by the presence of Sb^5+^ oxidized during calcination at high temperature.

### 3.2 Effect of grinding time on photocatalytic decomposition of acetaldehyde by STO:Rh,Sb

**Fig. 5:**
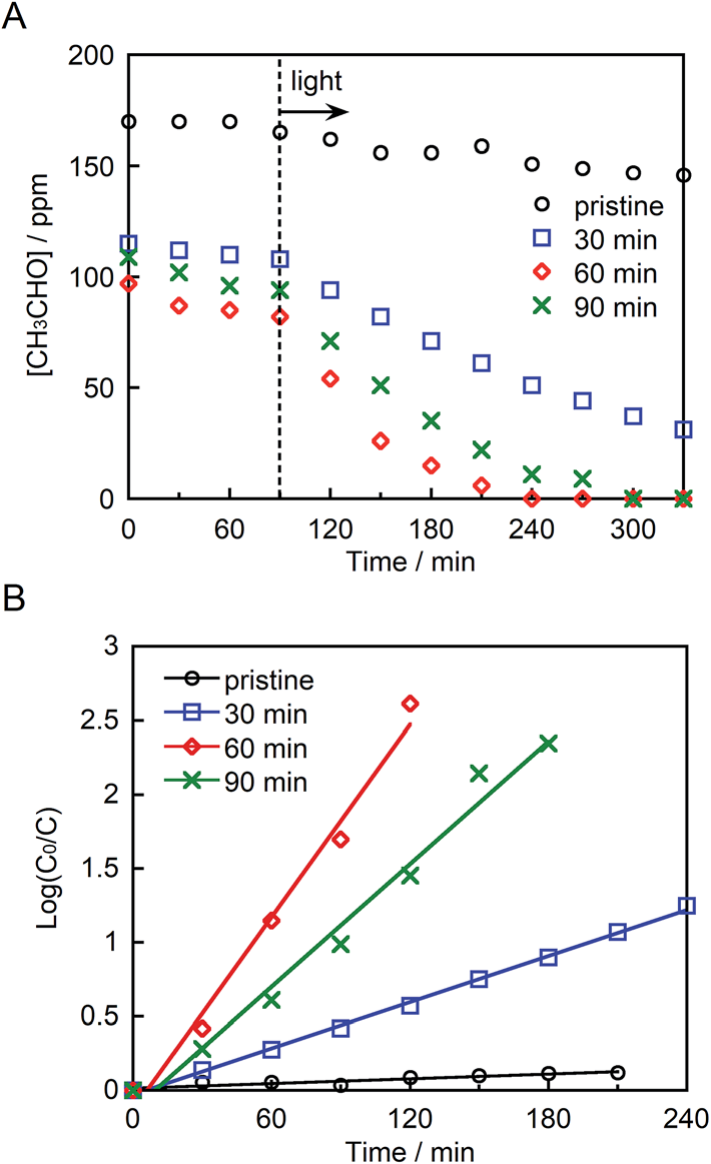
(A) Photocatalytic degradation of acetaldehyde and (B) linear kinetic fitting curves for pristine STO:Rh,Sb and STO:Rh,Sb ground for 30, 60, and 90 min under visible light irradiation (*λ* > 420 nm). Photocatalyst: 0.3 g, light source: 200 W Xe lamp with an L-42 filter cutoff filter (100 mW cm^−2^), initial acetaldehyde concentration: 150 ppm, gas phase volume: 500 mL.

Fig. 5A depicts the photocatalytic degradation of acetaldehyde by pristine and ground STO:Rh,Sb under visible light irradiation (*λ* > 420 nm). Before irradiation, the glass reaction vessel was kept in the dark, allowing the acetaldehyde to adsorb to the photocatalyst surface. Pristine STO:Rh,Sb (with small surface area) adsorbed negligible amounts of acetaldehyde, whereas ground STO:Rh,Sb adsorbed noticeable amounts. The acetaldehyde concentration drastically decreased in the presence of ground STO:Rh,Sb under visible light irradiation, whereas that of pristine STO:Rh,Sb decreased negligibly, suggesting that ball-milling effectively enhances the photocatalytic acetaldehyde degradation by STO:Rh,Sb. To determine the optimal grinding time, the logarithmic acetaldehyde concentration *versus* time was plotted (Fig. 4B). The reaction rate coefficient of STO:Rh,Sb ground for 30, 60, and 90 minutes, determined from the slopes of the linear graphs, were 0.52 × 10^−2^, 2.2 × 10^−2^, and 1.4 × 10^−2^ min^−1^, respectively, as shown in Table 1. Note that the rate coefficient was higher for STO:Rh,Sb ground for 60 min when compared with that for STO:Rh,Sb ground for 30 and 90 min. Therefore, grinding for 60 min achieved the highest photocatalytic activity of the STO:Rh,Sb. A large surface area enhances the photocatalytic performance by increasing the contact area between the target substances and the photocatalyst surface.^5^ The surface area of STO:Rh,Sb ground for 60 min was much larger than that of STO:Rh,Sb ground for 30 min. Therefore, grinding for 60 min showed higher photocatalytic activity than that for 30 min. On the other hand, grinding for 90 min showed lower photocatalytic activity than that for 60 min, despite the large surface area of the latter photocatalyst. As is well known, the number of crystal defects increases with increasing milling time.^54,55^ Crystal defects often work as recombination centers of excited electrons and holes, decreasing the photocatalytic performance. Therefore, grinding for 60 min achieved higher photocatalytic performance of the STO:Rh than grinding for 90 min.

**Table 1:** The rate coefficient of photocatalytic degradation of acetaldehyde over STO:Rh,Sb, ground STO:Rh,Sb and STO:Rh

Dopants	Milling time (min)	Rate coefficient × 10^−2^ (min^−1^)
Rh(1%),Sb(1%)	0	0.053
Rh(1%),Sb(1%)	30	0.52
Rh(1%),Sb(1%)	60	2.2
Rh(1%),Sb(1%)	90	1.4
Rh(0.1%),Sb(0.1%)	60	0.46
Rh(0.5%),Sb(0.5%)	60	2.1
Rh(2%),Sb(2%)	60	1.2
Rh(1%)	60	1.8

### 3.3 Optimal Rh and Sb doping amounts in STO:Rh,Sb for photocatalytic degradation of acetaldehyde

**Fig. 6:**
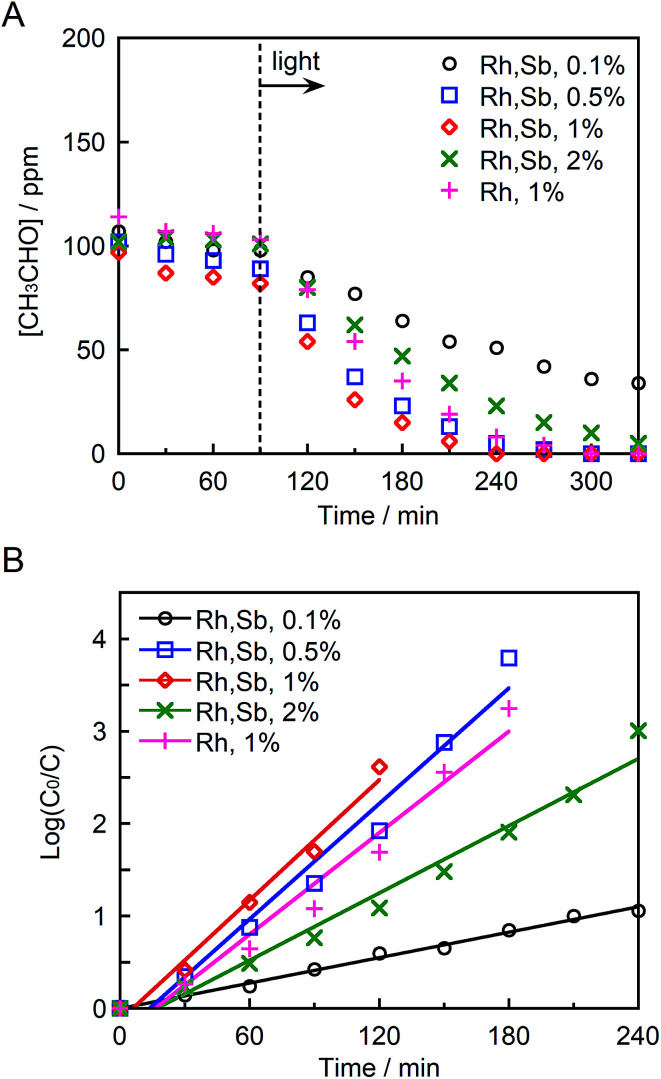
(A) Photocatalytic degradation of acetaldehyde and (B) linear kinetic fitting curves for STO:Rh(1%), STO:Rh(0.1%),Sb(0.1%), STO:Rh(0.5%),Sb(0.5%), STO:Rh(1%),Sb(1%), and STO:Rh(2%),Sb(2%) ground for 60 min under visible light irradiation (*λ* > 420 nm). Photocatalyst: 0.3 g, light source: 200 W Xe lamp with an L-42 filter cutoff filter (100 mW cm^−2^), initial acetaldehyde concentration: 150 ppm, gas phase volume: 500 mL.

Fig. 6A and B depict the photocatalytic degradation of acetaldehyde on actual and logarithmic scales of acetaldehyde concentration, respectively. Here, the Rh and Sb were doped at various concentrations, and they were photocatalytically activated by visible light irradiation. The reaction rate coefficient increased in the order (as shown in Table 1): STO:Rh(1%),Sb(1%) (2.2 × 10^−2^ min^−1^) > STO:Rh(0.5%),Sb(0.5%) (2.1 × 10^−2^ min^−1^) > STO:Rh(2%),Sb(2%) (1.2 × 10^−2^ min^−1^) > STO:Rh(0.1%),Sb(0.1%) (0.46 × 10^−2^ min^−1^). Clearly, STO:Rh(1%),Sb(1%) exhibited the highest photocatalytic activity among the prepared SrTiO_3_ photocatalysts. Increasing the doping amount not only enhances the absorption of visible light but also increases the number of recombination sites, thereby decreasing the photocatalytic performance. Therefore, the doping amount must be optimized for maximum photocatalytic degradation of organic substances. In addition, the co-doping effect by comparing the photocatalytic performances of ground STO:Rh(1%),Sb(1%) and ground STO:Rh(1%) was investigated. The reaction rate coefficient of ground STO:Rh(1%),Sb(1%) was higher than that of ground STO:Rh(1%) (2.2 × 10^−2^ min^−1^*vs.* 1.8 × 10^−2^ min^−1^), suggesting that antimony doping enhances the photocatalytic activity of STO:Rh. Furuhashi *et al.* revealed that antimony doping plays an important role in suppressing the formation of Rh^4+^ recombination centers.^53^ Thus, it is assumed that acetaldehyde is further adsorbed and oxidized on the active sites of the surface of STO:Rh,Sb during the photocatalytic reaction. This explains the efficient photocatalytic performance of the Sb-doped material. The conduction band level of STO:Rh,Sb is more negative than 0 V *vs.* the normal hydrogen electrode (NHE), whereas the electron donor level at which holes (Rh^3+^) are generated is *ca.* +2.1 V *vs.* NHE.^24^ Therefore, oxygen reduction (O_2_/HO_2_ = −0.13 V *vs.* NHE, O_2_/H_2_O_2_ = +0.68 V *vs.* NHE, O_2_/H_2_O = +1.23 V *vs.* NHE) and acetaldehyde oxidation should simultaneously proceed on the STO:Rh,Sb surface.^56,57^

**Fig. 7:**
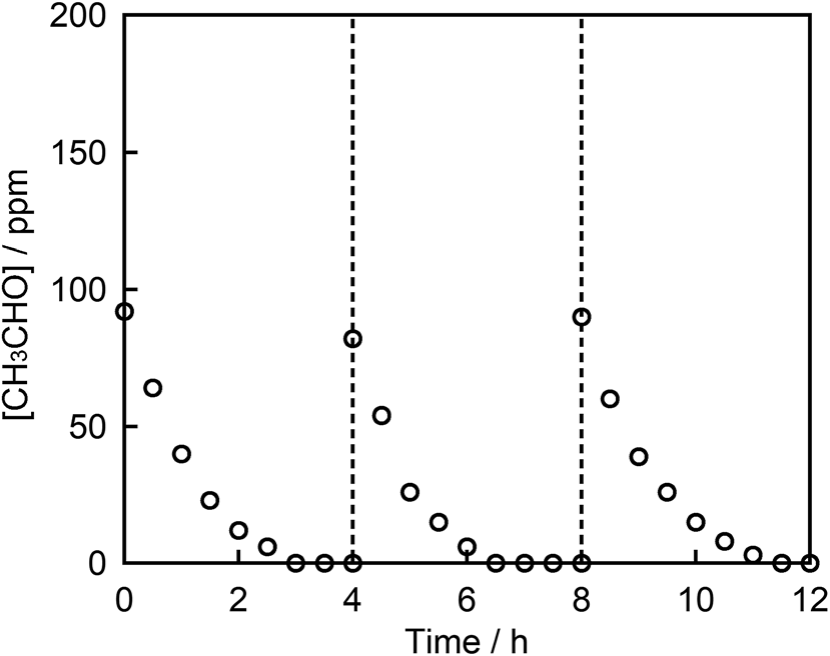
Photocatalytic degradation of acetaldehyde on STO:Rh(1%),Sb(1%) ground for 60 min under visible light irradiation (*λ* > 420 nm) over 3 cycles. Photocatalyst: 0.3 g, light source: 200 W Xe lamp with a L-42 filter cutoff filter (100 mW cm^−2^), initial acetaldehyde concentration: 150 ppm, gas phase volume: 500 mL.

Finally, the chemical stability of STO:Rh,Sb through three cycles of photocatalytic acetaldehyde decomposition by STO:Rh(1%),Sb(1%) ground for 60 min was investigated (Fig. 7). After each cycle, the organic compounds adsorbed to the photocatalyst during the photocatalytic reaction were removed by washing with ultrapure water. The photocatalytic degradation of acetaldehyde was very similar after the each cycles, indicating high photocatalytic stability of the STO:Rh,Sb sample. This result suggests that the photocatalyst is stable and reusable.

### 3.4 Carbon mass balance and cycling test of photocatalytic acetaldehyde degradation

The amounts of carbon dioxide and acetic acid generated after 4 h of the photocatalytic reaction over STO:Rh(1%),Sb(1%) ground for 60 min were determined to be 1.08 μmol (quantified by GC) and 2.87 μmol (quantified by HPLC), respectively. The carbon content in the acetic acid was 5.74 μmol (2.87 μmol × 2). Therefore, the total carbon content in the generated carbon dioxide and acetic acid was 6.82 μmol. The initial acetaldehyde concentration in the reaction was 150 ppm, corresponding to 6.70 μmol of carbon. Therefore, the carbon mass was almost balanced before and after the acetaldehyde degradation.

## 4. Conclusions

A visible-light-responsive STO:Rh,Sb photocatalyst that efficiently degrades acetaldehyde was successfully prepared. STO:Rh,Sb was synthesized by a solid-state reaction, following pulverization by ball-milling to increase the photocatalytic surface area. The optimal grinding time and doping amounts of Rh and Sb were also investigated. The highest photocatalytic activity was demonstrated by STO:Rh(1%),Sb(1%) ground for 60 min. Moreover, the photocatalytic performance of ground STO:Rh,Sb was higher than that of ground STO:Rh (doped without antimony) because antimony largely suppressed the formation of Rh^4+^, which works as a recombination center in STO:Rh. The ground STO:Rh,Sb also maintained its photocatalytic activity through three cycles, indicating that STO:Rh,Sb is stable and therefore reusable. Our work suggests that ground STO:Rh,Sb is potentially applicable to environmental remediation under visible light irradiation.

## Conflicts of interest

There are no conflicts to declare.

## Supplementary Material
